# The genome sequence of the Brown Litter Worm, *Bimastos eiseni* (Levinsen, 1884)

**DOI:** 10.12688/wellcomeopenres.21622.1

**Published:** 2024-05-20

**Authors:** Keiron D. Brown, Emma Sherlock, Liam M. Crowley, University of Oxford and Wytham Woods Genome Acquisition Lab, Darwin Tree of Life Barcoding collective, Wellcome Sanger Institute Tree of Life Management, Samples and Laboratory team, Wellcome Sanger Institute Scientific Operations: Sequencing Operations, Wellcome Sanger Institute Tree of Life Core Informatics team, Tree of Life Core Informatics collective, Darwin Tree of Life Consortium

**Affiliations:** 1 Biological Recording Company, Uxbridge, England, UK; 2 Natural History Museum, London, England, UK; 3 University of Oxford, Oxford, England, UK

**Keywords:** Bimastos eiseni, Brown Litter Worm, genome sequence, chromosomal, Haplotaxida

## Abstract

We present a genome assembly from an individual *Bimastos eiseni* (the Brown Litter Worm; Annelida; None; Haplotaxida; Lumbricidae). The genome sequence is 660.5 megabases in span. Most of the assembly is scaffolded into 17 chromosomal pseudomolecules. The mitochondrial genome has also been assembled and is 15.34 kilobases in length.

## Species taxonomy

Eukaryota; Opisthokonta; Metazoa; Eumetazoa; Bilateria; Protostomia; Spiralia; Lophotrochozoa; Annelida; Clitellata; Oligochaeta; Crassiclitellata; Lumbricina; Lumbricidae; Lumbricinae; *Bimastos*; *Bimastos eiseni* (Levinsen, 1884) (NCBI:txid320977).

## Background

**Figure 1.**
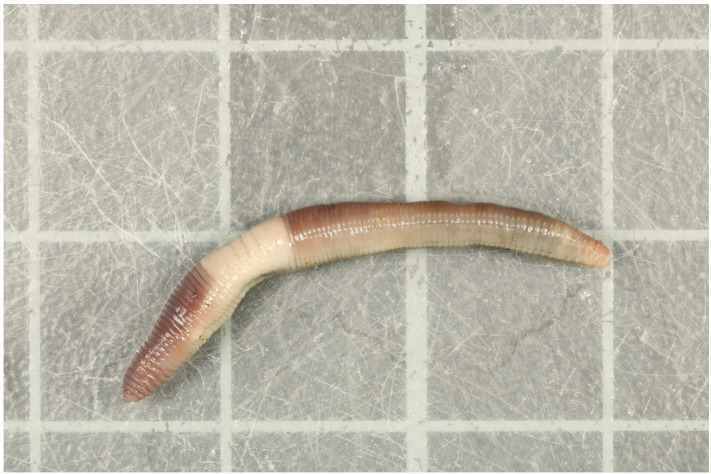
Photograph of a preserved specimen of *Bimastos eiseni* (not the specimen used for genome sequencing) taken by Keiron Derek Brown and Kerry Calloway.

*Bimastos eiseni* ( Figure 1) is identified using external morphological features, including the presence of a tanylobic head alongside a clitellum located on segments 24–27 to 32-–33 and no visible tubercula pubertatis ( Sherlock, 2018). Genetic studies have shown that this species is most likely North American in origin (though there are very few published records of this species on GBIF, GBIF Secretariat, 2024) and it should be considered non-native in Europe ( Csuzdi *et al.*, 2017). Despite this, *B. eiseni* is listed on the Global List of Introduced and Invasive Species Register for the USA and not on the lists for European territories where it is known to occur. *B. eiseni* has been recorded in England, Scotland and Ireland, but not yet recorded from Wales or Northern Ireland. The distribution is thought to be widespread through most of England, with sparse records in northern England, Scotland and Wales.

It is an epigeic (surface-dwelling) species of earthworm that is often associated with deadwood microhabitats, including tree hollows. It contributes towards the decomposition of waste plant material, contributing to waste recycling and detoxification ecosystem services ( Keith & Robinson, 2012).

We present a chromosomally complete genome sequence for *Bimastos eiseni*, based on one specimen from Wytham Woods, Oxfordshire, UK, as part of the Darwin Tree of Life Project. This project is a collaborative effort to sequence all named eukaryotic species in the Atlantic Archipelago of Britain and Ireland.

## Genome sequence report

The genome was sequenced from a specimen of *Bimastos eiseni* collected from Wytham Woods, Oxfordshire, UK (51.78, –1.34). A total of 32-fold coverage in Pacific Biosciences single-molecule HiFi long reads was generated. Primary assembly contigs were scaffolded with chromosome conformation Hi-C data. Manual assembly curation corrected 98 missing joins or mis-joins and removed 8 haplotypic duplications, reducing the scaffold number by 24.42%.

**Figure 2.**
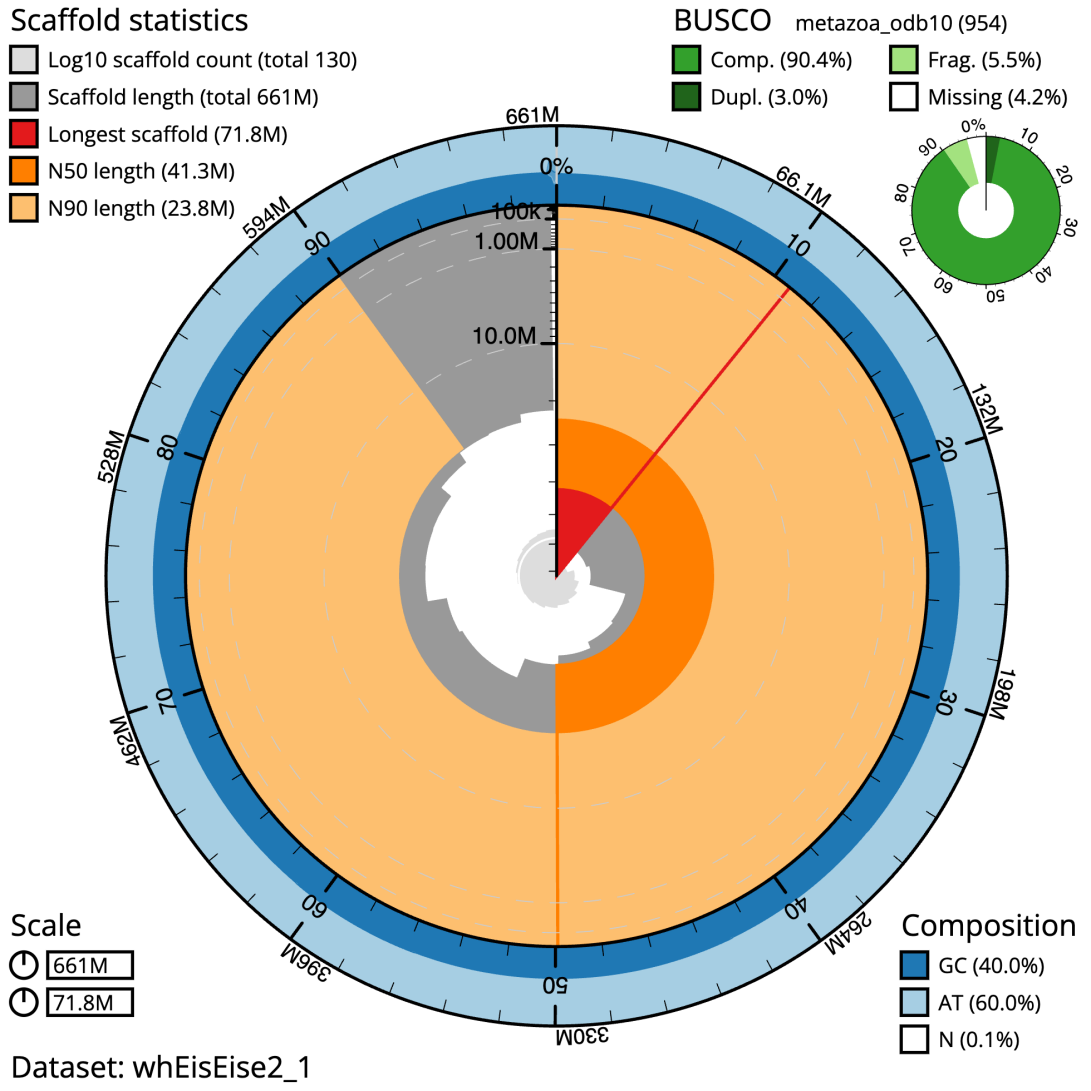
Genome assembly of *Bimastos eiseni*, whEisEise2.1: metrics. The BlobToolKit Snailplot shows N50 metrics and BUSCO gene completeness. The main plot is divided into 1,000 size-ordered bins around the circumference with each bin representing 0.1% of the 660,529,080 bp assembly. The distribution of scaffold lengths is shown in dark grey with the plot radius scaled to the longest scaffold present in the assembly (71,778,175 bp, shown in red). Orange and pale-orange arcs show the N50 and N90 scaffold lengths (41,312,700 and 23,769,983 bp), respectively. The pale grey spiral shows the cumulative scaffold count on a log scale with white scale lines showing successive orders of magnitude. The blue and pale-blue area around the outside of the plot shows the distribution of GC, AT and N percentages in the same bins as the inner plot. A summary of complete, fragmented, duplicated and missing BUSCO genes in the metazoa_odb10 set is shown in the top right. An interactive version of this figure is available at https://blobtoolkit.genomehubs.org/view/whEisEise2_1/dataset/whEisEise2_1/snail.

**Figure 3.**
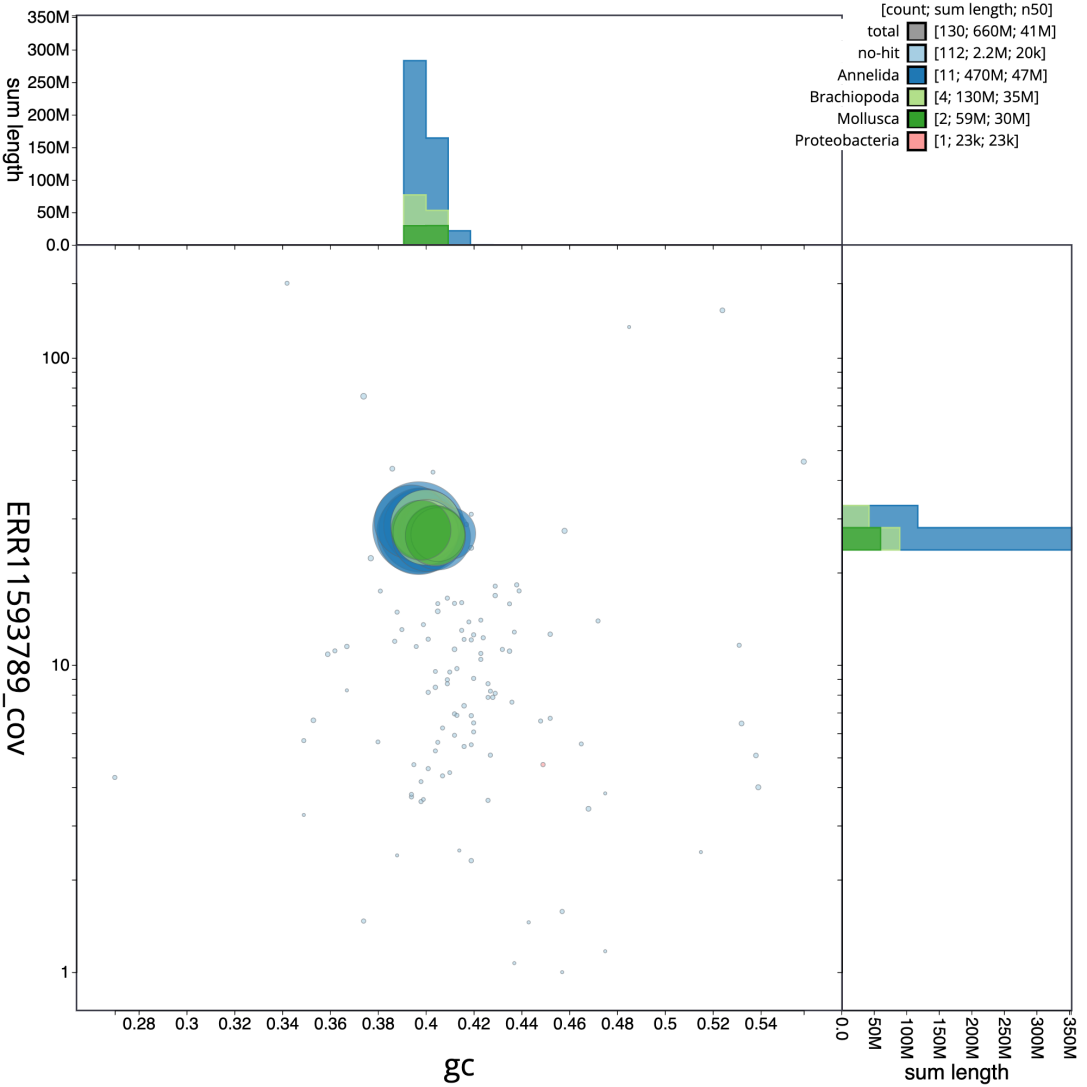
Genome assembly of *Bimastos eiseni*, whEisEise2.1: BlobToolKit GC-coverage plot. Scaffolds are coloured by phylum. Circles are sized in proportion to scaffold length. Histograms show the distribution of scaffold length sum along each axis. An interactive version of this figure is available at https://blobtoolkit.genomehubs.org/view/whEisEise2_1/dataset/whEisEise2_1/blob.

**Figure 4.**
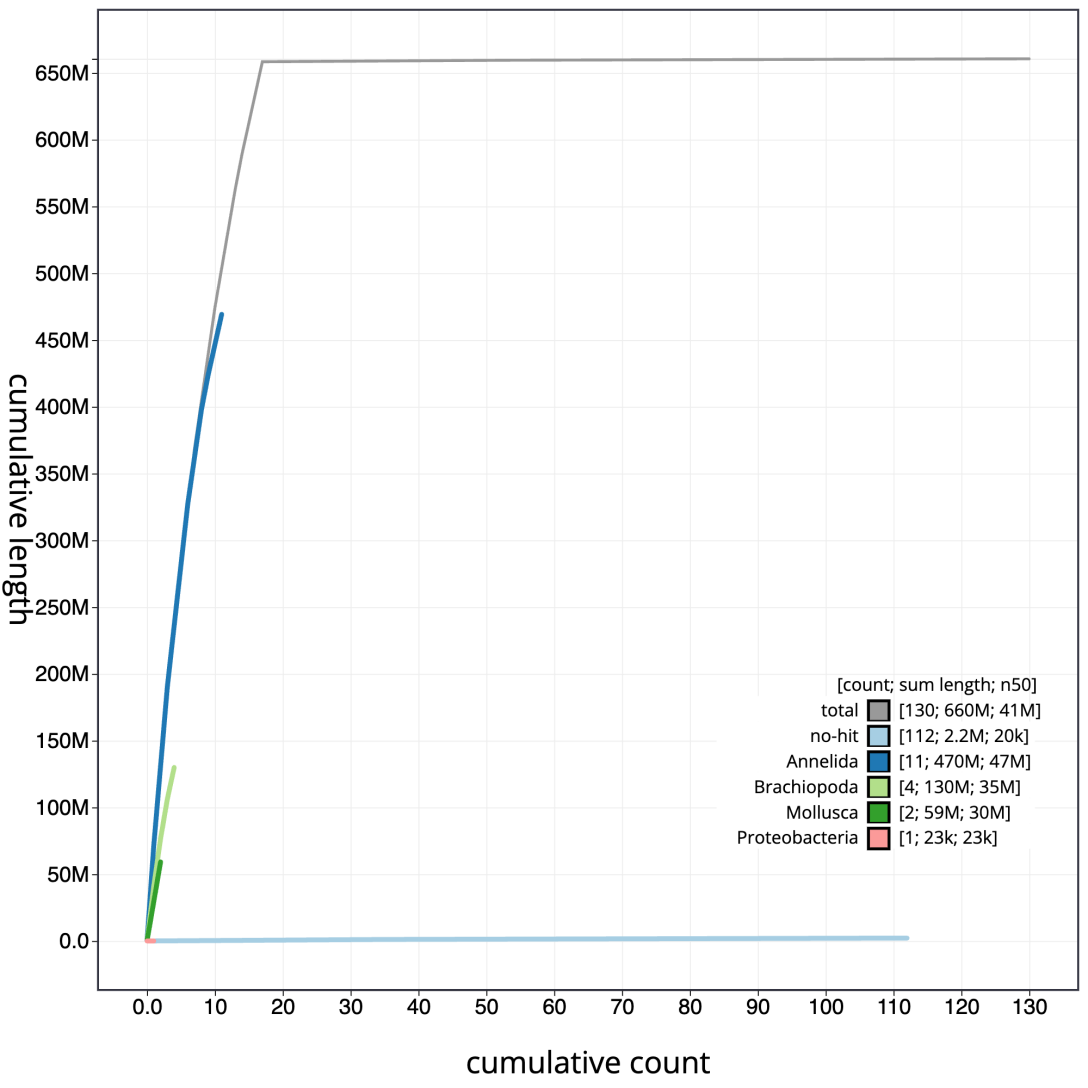
Genome assembly of *Bimastos eiseni*, whEisEise2.1: BlobToolKit cumulative sequence plot. The grey line shows cumulative length for all scaffolds. Coloured lines show cumulative lengths of scaffolds assigned to each phylum using the buscogenes taxrule. An interactive version of this figure is available at https://blobtoolkit.genomehubs.org/view/whEisEise2_1/dataset/whEisEise2_1/cumulative.

**Figure 5.**
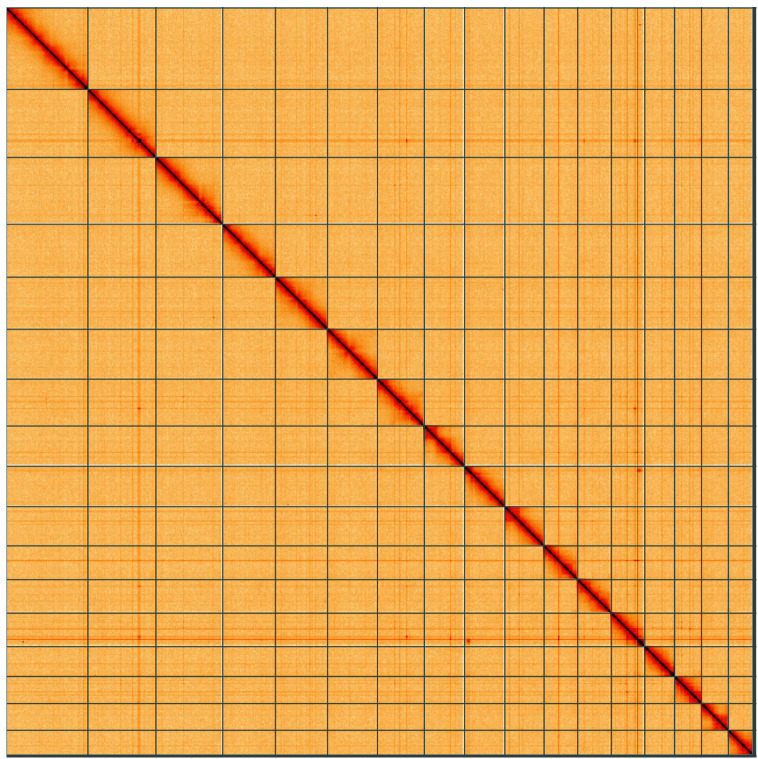
Genome assembly of *Bimastos eiseni*, whEisEise2.1: Hi-C contact map of the whEisEise2.1 assembly, visualised using HiGlass. Chromosomes are shown in order of size from left to right and top to bottom. An interactive version of this figure may be viewed at https://genome-note-higlass.tol.sanger.ac.uk/l/?d=eyFDKRI1QIeJbPX-y2JIPg.

The final assembly has a total length of 660.5 Mb in 129 sequence scaffolds with a scaffold N50 of 41.3 Mb ( Table 1). The snail plot in Figure 2 provides a summary of the assembly statistics, while the distribution of assembly scaffolds on GC proportion and coverage is shown in Figure 3. The cumulative assembly plot in Figure 4 shows curves for subsets of scaffolds assigned to different phyla. Most (99.66%) of the assembly sequence was assigned to 17 chromosomal-level scaffolds. Chromosome-scale scaffolds confirmed by the Hi-C data are named in order of size ( Figure 5; Table 2). While not fully phased, the assembly deposited is of one haplotype. Contigs corresponding to the second haplotype have also been deposited. The mitochondrial genome was also assembled and can be found as a contig within the multifasta file of the genome submission.

**Table 1.** Genome data for *Bimastos eiseni*, whEisEise2.1.

Project accession data		
Assembly identifier	whEisEise2.1	
Species	*Bimastos eiseni*	
Specimen	whEisEise2	
NCBI taxonomy ID	320977	
BioProject	PRJEB63410	
BioSample ID	SAMEA10166839	
Isolate information	whEisEise2 (DNA sequencing) whEisEise1 (Hi-C sequencing)	
Assembly metrics *		*Benchmark*
Consensus quality (QV)	57.4	*≥ 50*
*k*-mer completeness	99.99%	*≥ 95%*
BUSCO **	C:90.4%[S:87.3%,D:3.0%], F:5.5%,M:4.2%,n:954	*C ≥ 95%*
Percentage of assembly mapped to chromosomes	99.66%	*≥ 95%*
Sex chromosomes	None	*localised * *homologous pairs*
Organelles	Mitochondrial genome: 15.34 kb	*complete single * *alleles*
Raw data accessions		
PacificBiosciences SEQUEL II	ERR11593789	
Hi-C Illumina	ERR11606301	
Genome assembly		
Assembly accession	GCA_959347315.1	
*Accession of alternate haplotype*	GCA_959347335.1	
Span (Mb)	660.5	
Number of contigs	2277	
Contig N50 length (Mb)	0.5	
Number of scaffolds	129	
Scaffold N50 length (Mb)	41.3	
Longest scaffold (Mb)	71.78	

* Assembly metric benchmarks are adapted from column VGP-2020 of “Table 1: Proposed standards and metrics for defining genome assembly quality” from ( Rhie *et al.*, 2021). ** BUSCO scores based on the metazoa_odb10 BUSCO set using version 5.3.2. C = complete [S = single copy, D = duplicated], F = fragmented, M = missing, n = number of orthologues in comparison. A full set of BUSCO scores is available at https://blobtoolkit.genomehubs.org/view/whEisEise2_1/dataset/whEisEise2_1/busco.

**Table 2.** Chromosomal pseudomolecules in the genome assembly of *Bimastos eiseni*, whEisEise2.

INSDC accession	Chromosome	Length (Mb)	GC%
OY365806.1	1	71.78	39.5
OY365807.1	2	59.95	40.0
OY365808.1	3	58.85	39.5
OY365809.1	4	46.61	40.0
OY365810.1	5	45.96	39.5
OY365811.1	6	43.96	40.0
OY365812.1	7	41.31	40.0
OY365813.1	8	35.49	40.5
OY365814.1	9	35.47	40.0
OY365815.1	10	34.7	40.0
OY365816.1	11	29.72	40.5
OY365817.1	12	29.6	40.5
OY365818.1	13	29.5	40.0
OY365819.1	14	26.35	40.0
OY365820.1	15	23.77	40.0
OY365821.1	16	23.54	40.5
OY365822.1	17	21.78	41.0
OY365823.1	MT	0.02	34.5

The estimated Quality Value (QV) of the final assembly is 57.4 with *k*-mer completeness of 99.99%, and the assembly has a BUSCO v5.3.2 completeness of 90.4% (single = 87.3%, duplicated = 3.0%), using the metazoa_odb10 reference set ( *n* = 954).

Metadata for specimens, barcode results, spectra estimates, sequencing runs, contaminants and pre-curation assembly statistics are given at https://links.tol.sanger.ac.uk/species/320977.

## Methods

### Sample acquisition and nucleic acid extraction

Specimens of *Bimastos eiseni* were collected from Wytham Woods, Oxfordshire (biological vice-county Berkshire), UK (latitude 51.78, longitude –1.34) on 2021-05-27. The specimens were collected and identified by Keiron Brown (University of Oxford), with confirmation of species by Emma Sherlock (Natural History Museum), and snap-frozen on dry ice. A specimen with ID Ox001367 (ToLID whEisEise2) was used for DNA sequencing and a specimen with ID Ox001365 (ToLID whEisEise1) was used for Hi-C sequencing.

The workflow for high molecular weight (HMW) DNA extraction at the Wellcome Sanger Institute (WSI) includes a sequence of core procedures: sample preparation; sample homogenisation, DNA extraction, fragmentation, and clean-up. In sample preparation, the whEisEise2 sample was weighed and dissected on dry ice ( Jay *et al.*, 2023). Tissue from the posterior body was homogenised using a PowerMasher II tissue disruptor ( Denton *et al.*, 2023a). 

HMW DNA was extracted using the Automated MagAttract v1 protocol ( Sheerin *et al.*, 2023). DNA was sheared into an average fragment size of 12–20 kb in a Megaruptor 3 system with speed setting 30 ( Todorovic *et al.*, 2023). Sheared DNA was purified by solid-phase reversible immobilisation ( Strickland *et al.*, 2023): in brief, the method employs a 1.8X ratio of AMPure PB beads to sample to eliminate shorter fragments and concentrate the DNA. The concentration of the sheared and purified DNA was assessed using a Nanodrop spectrophotometer and Qubit Fluorometer and Qubit dsDNA High Sensitivity Assay kit. Fragment size distribution was evaluated by running the sample on the FemtoPulse system.

Protocols developed by the WSI Tree of Life laboratory are publicly available on protocols.io ( Denton *et al.*, 2023b).

### Sequencing

Pacific Biosciences HiFi circular consensus DNA sequencing libraries were constructed according to the manufacturers’ instructions. DNA sequencing was performed by the Scientific Operations core at the WSI on a Pacific Biosciences SEQUEL II instrument. Hi-C data were also generated from posterior body tissue of whEisEise1 using the Arima2 kit and sequenced on the Illumina NovaSeq 6000 instrument.

### Genome assembly, curation and evaluation

Assembly was carried out with Hifiasm ( Cheng *et al.*, 2021) and haplotypic duplication was identified and removed with purge_dups ( Guan *et al.*, 2020). The assembly was then scaffolded with Hi-C data ( Rao *et al.*, 2014) using YaHS ( Zhou *et al.*, 2023). The assembly was checked for contamination and corrected as described previously ( Howe *et al.*, 2021). Manual curation was performed using HiGlass ( Kerpedjiev *et al.*, 2018) and Pretext ( Harry, 2022). The mitochondrial genome was assembled using MitoHiFi ( Uliano-Silva *et al.*, 2023), which runs MitoFinder ( Allio *et al.*, 2020) or MITOS ( Bernt *et al.*, 2013) and uses these annotations to select the final mitochondrial contig and to ensure the general quality of the sequence.

A Hi-C map for the final assembly was produced using bwa-mem2 ( Vasimuddin *et al.*, 2019) in the Cooler file format ( Abdennur & Mirny, 2020). To assess the assembly metrics, the *k*-mer completeness and QV consensus quality values were calculated in Merqury ( Rhie *et al.*, 2020). This work was done using Nextflow ( Di Tommaso *et al.*, 2017) DSL2 pipelines “sanger-tol/readmapping” ( Surana *et al.*, 2023a) and “sanger-tol/genomenote” ( Surana *et al.*, 2023b). The genome was analysed within the BlobToolKit environment ( Challis *et al.*, 2020) and BUSCO scores ( Manni *et al.*, 2021; Simão *et al.*, 2015) were calculated.

Table 3 contains a list of relevant software tool versions and sources.

**Table 3.** Software tools: versions and sources.

Software tool	Version	Source
BlobToolKit	4.2.1	https://github.com/blobtoolkit/blobtoolkit
BUSCO	5.3.2	https://gitlab.com/ezlab/busco
Hifiasm	0.19.5-r587	https://github.com/chhylp123/hifiasm
HiGlass	1.11.6	https://github.com/higlass/higlass
Merqury	MerquryFK	https://github.com/thegenemyers/MERQURY.FK
MitoHiFi	3	https://github.com/marcelauliano/MitoHiFi
PretextView	0.2	https://github.com/wtsi-hpag/PretextView
purge_dups	1.2.5	https://github.com/dfguan/purge_dups
sanger-tol/genomenote	v1.0	https://github.com/sanger-tol/genomenote
sanger-tol/readmapping	1.1.0	https://github.com/sanger-tol/readmapping/tree/1.1.0
YaHS	1.2a.2	https://github.com/c-zhou/yahs

### Wellcome Sanger Institute – Legal and Governance

The materials that have contributed to this genome note have been supplied by a Darwin Tree of Life Partner. The submission of materials by a Darwin Tree of Life Partner is subject to the **‘Darwin Tree of Life Project Sampling Code of Practice’**, which can be found in full on the Darwin Tree of Life website here. By agreeing with and signing up to the Sampling Code of Practice, the Darwin Tree of Life Partner agrees they will meet the legal and ethical requirements and standards set out within this document in respect of all samples acquired for, and supplied to, the Darwin Tree of Life Project. 

Further, the Wellcome Sanger Institute employs a process whereby due diligence is carried out proportionate to the nature of the materials themselves, and the circumstances under which they have been/are to be collected and provided for use. The purpose of this is to address and mitigate any potential legal and/or ethical implications of receipt and use of the materials as part of the research project, and to ensure that in doing so we align with best practice wherever possible. The overarching areas of consideration are:

• Ethical review of provenance and sourcing of the material

• Legality of collection, transfer and use (national and international) 

Each transfer of samples is further undertaken according to a Research Collaboration Agreement or Material Transfer Agreement entered into by the Darwin Tree of Life Partner, Genome Research Limited (operating as the Wellcome Sanger Institute), and in some circumstances other Darwin Tree of Life collaborators.

## Data Availability

European Nucleotide Archive: *Bimastos eiseni*. Accession number PRJEB63410; https://identifiers.org/ena.embl/PRJEB63410 ( Wellcome Sanger Institute, 2023). The genome sequence is released openly for reuse. The Bimastos eiseni genome sequencing initiative is part of the Darwin Tree of Life (DToL) project. All raw sequence data and the assembly have been deposited in INSDC databases. The genome will be annotated using available RNA-Seq data and presented through the Ensembl pipeline at the European Bioinformatics Institute. Raw data and assembly accession identifiers are reported in Table 1.

## Author information

Members of the University of Oxford and Wytham Woods Genome Acquisition Lab are listed here: https://doi.org/10.5281/zenodo.7125292.

Members of the Darwin Tree of Life Barcoding collective are listed here: https://doi.org/10.5281/zenodo.4893703.

Members of the Wellcome Sanger Institute Tree of Life Management, Samples and Laboratory team are listed here: https://doi.org/10.5281/zenodo.10066175.

Members of Wellcome Sanger Institute Scientific Operations: Sequencing Operations are listed here: https://doi.org/10.5281/zenodo.10043364.

Members of the Wellcome Sanger Institute Tree of Life Core Informatics team are listed here: https://doi.org/10.5281/zenodo.10066637.

Members of the Tree of Life Core Informatics collective are listed here: https://doi.org/10.5281/zenodo.5013541.

Members of the Darwin Tree of Life Consortium are listed here: https://doi.org/10.5281/zenodo.4783558.
