# Supplementary material for: The genome sequence of the Brown Litter Worm, Bimastos eiseni (Levinsen, 1884)
Source: Wellcome Open Res. 2024 May 20;9:279. [Version 1] doi: 10.12688/wellcomeopenres.21622.1 (PMC11408909; doi:10.12688/wellcomeopenres.21622.1)
Supplement: Peer review history

# Reviewer report: Reviewer response for version 1

**DOI:** 10.21956/wellcomeopenres.23894.r94241

**Published:** 2024-09-17

**Authors:** Natasha Tilikj

**Affiliations:** 1 Complutense University of Madrid, Madrid, Spain

Brown et al. have submitted a manuscript detailing the genomic assembly of the Brown Litter Worm, an epigeic species with a broad distribution across much of England. Using long-read sequencing and Hi-C data, they have produced a genome of exceptional quality, as demonstrated by the assembly metrics. Although my expertise in genome conformation techniques is somewhat limited, I found the results and the chromosomal visualization in Figure 5 to be clearly presented and easy to follow.

 Given the diversity in earthworm sizes, it would be beneficial to include a scale bar in Figure 1 to provide clearer context. Additionally, once genome annotation becomes available, it would be valuable for the authors to comment on the mitochondrial genome, noting any significant differences between it and the whole genome. This could offer interesting insights and further enrich the study.

Are sufficient details of methods and materials provided to allow replication by others?

Yes

Is the rationale for creating the dataset(s) clearly described?

Yes

Are the datasets clearly presented in a useable and accessible format?

Yes

Are the protocols appropriate and is the work technically sound?

Yes

Reviewer Expertise:

Zoology, Transcriptomics, Climate change

I confirm that I have read this submission and believe that I have an appropriate level of expertise to confirm that it is of an acceptable scientific standard.

# Reviewer report: Reviewer response for version 1

**DOI:** 10.21956/wellcomeopenres.23894.r92905

**Published:** 2024-08-23

**Authors:** Dong Zhang

**Affiliations:** 1 Lanzhou University, Lanzhou, Gansu, China; 2 Tibet University, Lhasa, China

The paper is well-written, with a high-quality genome that is thoroughly analyzed and presented. The methodology is sound, and I found the Methods section particularly informative. However, I have one concern: the genome appears to lack structural annotation. Completing the annotation work would greatly enhance the manuscript and should be considered before indexing.

Are sufficient details of methods and materials provided to allow replication by others?

Yes

Is the rationale for creating the dataset(s) clearly described?

Yes

Are the datasets clearly presented in a useable and accessible format?

Yes

Are the protocols appropriate and is the work technically sound?

Yes

Reviewer Expertise:

genome

I confirm that I have read this submission and believe that I have an appropriate level of expertise to confirm that it is of an acceptable scientific standard, however I have significant reservations, as outlined above.

# Reviewer report: Reviewer response for version 1

**DOI:** 10.21956/wellcomeopenres.23894.r94243

**Published:** 2024-08-22

**Authors:** José M Martín-Durán

**Affiliations:** 1 Queen Mary University of London, London, UK

This genome note reports the sequencing and assembly of the genome of the earthworm *Bimastos eiseni*, a species thought to be invasive in the US but that might actually come from there and be non-native in Europe. *Bimastos eiseni *is an important ecological player, as it is often associated with the decomposition of organic matter in soils. A high-quality genome will help with elucidating the origin of this species and studies on their ecological role.

 Strengths:

- High-quality genome assembly and important resource for a potentially invasive species.

Weaknesses:

- Taxonomical category "None" in the abstract should be amended. This belongs to Clitellata.
- Correct typo in the second line of the background (extra - between 32 and 33).
- Annotation not available, but this will be sorted in the future.

Are sufficient details of methods and materials provided to allow replication by others?

Yes

Is the rationale for creating the dataset(s) clearly described?

Yes

Are the datasets clearly presented in a useable and accessible format?

Yes

Are the protocols appropriate and is the work technically sound?

Yes

Reviewer Expertise:

Evolutionary biology, developmental biology, comparative genomics

I confirm that I have read this submission and believe that I have an appropriate level of expertise to confirm that it is of an acceptable scientific standard.
